# Turning Good Intentions Into Actions by Using the Health Action Process Approach to Predict Adherence to Internet-Based Depression Prevention: Secondary Analysis of a Randomized Controlled Trial

**DOI:** 10.2196/jmir.8814

**Published:** 2018-01-11

**Authors:** Anna-Carlotta Zarski, Matthias Berking, Dorota Reis, Dirk Lehr, Claudia Buntrock, Ralf Schwarzer, David Daniel Ebert

**Affiliations:** ^1^ Friedrich-Alexander-University Erlangen-Nürnberg Erlangen Germany; ^2^ Leuphana University Lüneburg Lüneburg Germany; ^3^ University Koblenz-Landau Landau Germany; ^4^ SWPS University of Social Sciences and Humanities Warszawa Poland

**Keywords:** health action process approach, adherence, Internet intervention, depression prevention

## Abstract

**Background:**

Many individuals engaging in Internet-based interventions fail to complete these treatments as intended. The processes responsible for treatment adherence in Internet-based interventions are still poorly understood.

**Objective:**

The aim of this study was to investigate to what extent adherence in an Internet-based intervention can be predicted by motivational and volitional factors outlined in the health action process approach (HAPA).

**Methods:**

This study investigated motivational and volitional factors included in HAPA in a randomized controlled trial to predict treatment adherence of N=101 individuals with subclinical depression in the intervention group of a depression prevention intervention (GET.ON Mood Enhancer). Adherence was operationalized as the number of completed treatment modules. Using longitudinal structural equation modeling, HAPA variables (motivational, maintenance, and recovery self-efficacy, outcome expectancies, intention, and planning) were assessed at baseline and their associations with adherence 7 weeks later.

**Results:**

Planning predicted adherence. Better planning was, in turn, associated with higher levels of maintenance self-efficacy, and the latter significantly affected treatment adherence via planning. The other hypothesized direct associations were not significant. In total, the HAPA variables accounted for 14% of variance in treatment adherence.

**Conclusions:**

Planning emerged as the strongest predictor of treatment adherence in highly motivated participants in an Internet-based intervention out of all HAPA variables investigated. Findings are in line with the hypothesis that planning facilitates the translation of good intentions into actions. The findings imply that systematically fostering planning skills and maintenance self-efficacy prior to or during Internet-based interventions would help participants to successfully complete these treatments.

**Trial Registration:**

German Clinical Trials Register DRKS00005973; https://www.drks.de/drks_web/navigate.do? navigationId=trial.HTML&TRIAL_ID=DRKS00005973 (Archived by WebCite at http://www.webcitation.org/6uxCy64sy).

## Introduction

Internet-based interventions have been shown to prevent the incidence of mental health disorders such as depression or anxiety and reduce associated symptom severity [1,2]. Internet-based interventions used to prevent mental health disorders have several advantages. First, they are readily accessible at any time and place. Second, individuals can choose to remain anonymous and thus avoid stigmatization. Third, individuals tend to have more active roles in (guided) self-help interventions, and for this reason, it might be easier for them to integrate the newly acquired skills in their day-to-day lives. Fourth, individuals can work at their own pace and go through materials as often as they want [3].

The high autonomy and flexibility of Internet-based interventions facilitate low-threshold access to treatment but also place high self-regulatory demands on participants and thereby entice treatment cessation [4]. Low treatment adherence rates can, in turn, reduce the effectiveness of Internet-based interventions substantially [5,6]. Many individuals struggle to begin or complete Internet-based interventions despite being highly motivated. This suggests that good intentions do not guarantee sustained adherence or address how to adequately deal with barriers associated with Internet-based interventions such as allocating time to work on the training modules [7]. It also shows the need to clarify which factors and processes determine whether participants can put their intentions into action in order to complete Internet-based interventions.

Concerning sociodemographic characteristics and symptom severity and their influence on treatment adherence, few clear predictors have emerged. Female gender, for example, has been shown to be a predictor of higher treatment adherence in contrast to, for example, level of education, marital status, employment, or ethnicity, which have not been found to be associated with adherence [8]. For age and baseline symptom severity, the results were inconsistent [8]. The findings of previous studies can, however, explain the variance in adherence only to some extent [9-12], with findings of, inter alia, 11% [13]. There is empirical evidence showing that the intrinsic motivation to commence an intervention, treatment expectancy, and the ability to focus on future goals influence adherence and attrition in Internet-based interventions [7,14-16]. However, only a few studies used a theoretical framework to explain adherence in Internet-based intervention [14].

**Figure 1:**
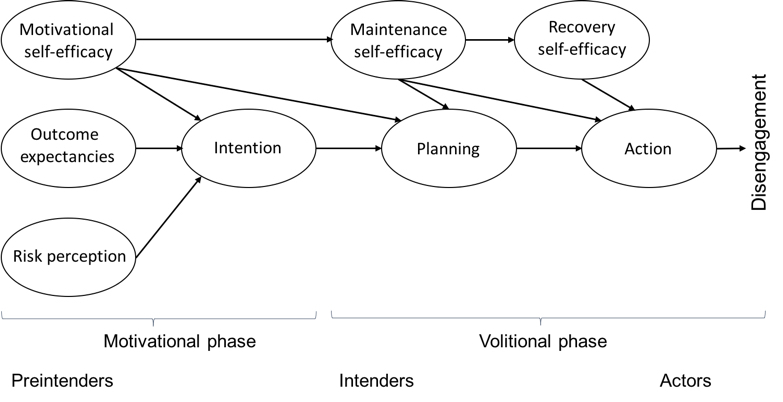
The health action process approach model.

As suggested by the theoretical framework of the health action process approach (HAPA), the gap between intention and behavior can be explained by volitional factors [17,18]. Describing 2 phases and 3 stages, HAPA can explain why individuals adopt and maintain a wide range of health behaviors [17,19]. As suggested by this model, a motivational phase, in which the intention to adopt a certain kind of health behavior is developed, is followed by a volitional phase, in which behavior is planned, prepared, and executed (see Figure 1) [20]. HAPA also assumes that individuals pass through different stages such as preintention, intention, and action when adopting new behavior [21,22]. In the motivational phase, individuals are seen as preintenders if they have not yet decided to adopt a new behavior. In the volitional phase, individuals are classified as either intenders who have decided to adopt the target behavior or as actors already performing the behavior [21,23].

The influence of variables of the motivational and volitional phases on health behavior depends on the stage individuals are currently in (see Figure 1) [24,25]. In the motivational phase, to develop an intention is assumed to be influenced by outcome expectancies, risk perceptions, and motivational self-efficacy [26-29]. Outcome expectancies are a person’s positive and negative expectations concerning the consequences of adopting a behavior, and they have been shown to be the strongest predictor in the motivational phase [20].

Risk perception is a measure of perceived vulnerability in terms of health impairment, and compared to outcome expectancies, this has shown to be a weaker predictor of intention [20,26,30]. Motivational self-efficacy, which can be defined as the belief in one’s ability to perform the targeted behavior, is regarded as the second best predictor of behavioral intentions [27]. By expressing an explicit behavioral intention, individuals are motivated to act, although such motivation does not necessarily need to be translated into actual behaviors, given the likelihood that barriers emerge rendering the intention instable. Thus, an intention may be seen as a distal antecedent of action. It is assumed that initial motivation at the onset of an intervention makes a difference for all subsequent processes, thus, influencing the likelihood of planning as well as the eventual success of the intervention as reflected by higher adherence levels.

In the volitional phase, individuals initiate and maintain the target behavior [31]. In this phase, maintenance self-efficacy and recovery self-efficacy have shown to be crucial for individuals facing imminent barriers and for those coming to terms with relapses, respectively [32,33]. Moreover, planning has been shown, in line with HAPA, to be a mediator between intentions and behaviors and to further facilitate the translation of intentions into actions [30,34-37]. Planning is regarded as a prospective self-regulatory skill where an individual specifies the situational context in which one will enact to ensure that behavioral performance is achieved. Planning requires a mental representation of how to achieve some future outcome that allows the individual to link the intended behavior with a particular context for its enactment, thus connecting the individual with good opportunities to act. Planning may also include the anticipation of barriers and the generation of alternative behaviors to overcome those [38].

Currently, it is unclear to what extent motivational and volitional variables included in HAPA can also explain the intention-action gap in the field of adherence in Internet-based interventions. To shed light on these factors and processes in Internet-based interventions, this study explores the intention-behavior gap by assessing motivational and volitional adherence predictors based on the HAPA model for individuals showing subclinical symptoms of depression but not fulfilling the criteria for a major depressive disorder. Treatment adherence is operationalized here by the number of completed treatment modules. First, according to HAPA, it can be expected that differences in treatment adherence between the participants are mainly due to volitional factors such as planning because participants can already be classified as intenders due to their decision to take part in an Internet-based intervention for depression prevention [21]. Accordingly, motivational self-efficacy and outcome expectancies should not predict intention. Second, it was hypothesized that higher levels of planning, maintenance, and recovery self-efficacy in the volitional phase should explain higher rates of treatment adherence rates in this sample. Hypotheses were tested using a structural equation model based on a longitudinal research design.

## Methods

The study outlined below was described in greater detail elsewhere [39]. Data for the secondary analyses were collected in a randomized controlled trial (RCT) evaluating a guided Internet-based intervention for depression prevention (GET.ON Mood Enhancer) comparing an intervention group to a waitlist control group. Participants assigned to the waitlist control group did not have access to the intervention during the first 3 months after randomization. For the analyses in this study, assessment took place at baseline (T1) and at posttreatment 7 weeks after randomization (T2). The study was approved by the medical ethics committee of the Leuphana University of Lüneburg (reference number: Ebert201404_Depr) and registered with the German Clinical Trials Register [DRKS00005973].

### Sample

The analyses were conducted using the intervention group sample (n=102), which was given access to the Internet-based intervention directly after randomization. One participant of the intervention group was excluded because of missing data in the HAPA questionnaire at baseline, resulting in a sample of n=101 for this analysis. Applicants were included in the study if they (1) had a subthreshold depression (Center for Epidemiological Studies Depression Scale [CES-D] ≥16), (2) were 18 years or older, (3) had Internet access, (4) were willing to give informed consent, and (5) did not show a notable suicidal risk (Beck Depression Inventory item 9 >1) [40-42]. Exclusion criteria were (1) a current major depressive episode as defined by the *Diagnostic and Statistical Manual of Mental Disorders, Fourth Edition, Text Revision* criteria assessed with the Structured Clinical Interview for Diagnostic and Statistical Manual of Mental Disorders (Fourth Edition) Axis I Disorders [43], (2) a major depressive episode in the past 6 months, (3) bipolar disorder, (4) psychotic disorder, (5) currently receiving psychotherapy or having received psychotherapy for any kind of mental health disorder in the past 6 months, or (6) being on a waiting list for psychotherapy for any kind of mental health problems. All applicants could use routine care (eg, they could visit their general practitioners).

### Procedure

Potential participants were recruited from the general population with the help of a large German health insurance company (Barmer Gmünder Ersatzkasse), and the study was also announced in newspaper articles, on-air media, and related websites. Individuals interested in participating in the study applied online on the website designed for this study (www.geton-training.de) by submitting their email address or by sending an email to the research team. Applicants then received an information letter via email with detailed information on the intervention and the study. In this letter, they were informed that they could withdraw from the intervention or study at any time without any negative consequences. Applicants were then screened for study inclusion and exclusion criteria, and those who met all of the criteria considered during this screening process were scheduled for the semistructured clinical interview conducted by telephone [44,45]. Individuals who met none of the exclusion criteria after the interviews, completed the baseline assessment, and returned the informed consent form via mail or email entered the study. They were then randomly allocated to either the intervention group or the waitlist control group. Balanced block randomization took place at an individual level in blocks of 12 to maintain a ratio of 1:1 between the 2 study groups. This process was completed using an automated computer-based random numbers table and completed by a researcher not involved in the study.

### Intervention

The intervention was described in greater detail by Buntrock and her colleagues [1,39,46]. It is based on behavior therapy and problem-solving therapy and consists of 6 modules and 1 booster session. Additional elective modules integrated in the last 3 sessions are directed at sleep hygiene, relaxation techniques, and dealing with worrying thoughts. Each session can be completed in approximately 30 to 60 minutes. Participants were advised to attend a maximum of 2 sessions per week but at least 1. Consequently, the training takes about 3 to 6 weeks plus a booster session 4 weeks after the end of the training. Lessons consist of general text-based information, testimonials, interactive elements such as exercises, and other content such as mp3 audio files, video clips, and downloadable work sheets. The intervention was conceptualized as guided self-help, and intervention elements such as self-gratification were included to support participants during the self-help process and encourage them to continue treatment. The training was gradually adjusted to the specific needs of individual participants based on their responses to and choices of different options. Participants were encouraged to keep a daily online training diary to monitor their mood and reflect on mood-enhancing activities. One key feature of the intervention is the focus on homework assignments, which allowed participants to integrate newly acquired coping skills and techniques into their daily lives. A secure Web-based platform (Advanced Encryption Standard [AES] 256-bit encryption) was used for the training. Participants accessed the intervention on the platform using their email addresses and passwords that they had created. If desired, participants received a set of about 42 standardized automatic motivational text messages including descriptions of short exercises on their mobile phones.

### Adherence-Focused Guidance

To support their adherence to the training, participants received guidance by an electronic coach (eCoach) using an adherence-focused guidance concept, described in detail elsewhere [12,47]. Adherence-focused guidance consisted of adherence monitoring and feedback on demand. Adherence monitoring included checking module completion on a regular basis and sending reminders in case participants had not completed at least 1 module within 7 days. Feedback on demand included giving participants the opportunity to contact the eCoach and receive individual support or feedback on training modules within 48 hours. Only a few participants (6/101, 5.9%) requested feedback, resulting in 15 instances of content feedback for the entire sample. This corresponds to an average of 0.15 feedback demands per participant (range 0-5, standard deviation [SD] 0.71). Checking module completion and providing reminders, then, accounted for most of the time spent per participant. The eCoaches were trained psychologists who followed feedback guidelines defined in the standardized manual for the intervention, in accordance with the supportive accountability model [48]. The supportive accountability model assumes that human support in the context of Internet-based interventions increases adherence rates because participants tend to develop a sense of commitment toward an eCoach, who is perceived as trustworthy, benevolent, and knowledgeable.

### Measures

Self-report measures for the present analyses were collected at baseline and at posttreatment also using the secured online-based assessment system (AES 256-bit encrypted).

#### Sociodemographic Information and Depression

Data on sociodemographic information and depression were collected at baseline. Depressive symptom severity was measured by the self-report CES-D, the clinician-rated Hamilton Rating Scale for Depression (HRSD24), and the Quick Inventory of Depressive Symptomatology–Clinician Rating (QIDS-CR16). A cutoff on the CES-D of 23 is regarded as an indicator of clinically relevant depressive symptoms in German samples [42,49]. The cutoff points of 10, 19, 27, and 35 of the HRSD24 indicate mild, moderate, severe, and very severe depression, respectively [50-52]. The QIDS cutoff points of 6, 11, 16, and 21 represent the thresholds for mild, moderate, severe, and very severe depression, respectively [50].

#### Health Action Process Approach Measures

The HAPA questionnaire, designed in accordance with the guidelines prepared by Schwarzer [19], was completed at baseline. All items were measured on a 4-point Likert scale ranging from 1=not true at all to 4=exactly true.

Motivational self-efficacy regarding the capability of the participants to complete the training modules including the exercises was measured with 2 items. One item was “I am confident that I am able to complete all 6 modules of the online training and the booster session 4 weeks after completion of the training even if there might be problems.” The Spearman-Brown reliability estimate was reported for the 2-item scale and showed excellent internal consistency (*r*_s_=.91) in this study.

Outcome expectancies were assessed with 2 items measuring the subjective beliefs concerning the positive impact of training adherence on mental health outcomes. One of the items used here was “If I complete 1 module of the online training per week, I will become more resilient in my everyday life.” The Spearman-Brown reliability estimate for this scale showed adequate internal consistency (*r*_s_=.66).

Intention was assessed with 1 item asking participants to what extent they intend to complete all 6 modules of the online training and the booster session 4 weeks after completing the training.

Planning was measured with 4 items, and this variable was used to assess whether participants have made concrete plans when and how they will complete the training, also in case of potential difficulties. One of the items was “I have already made detailed plans how often I will work on the modules during the week.” In this study, Cronbach alpha was found to be excellent (alpha=.92).

Maintenance self-efficacy was measured with 4 items focusing on potential obstacles during later stages of the online training. Barriers considered in here were no immediate positive effects, technical problems, perceived difficulties, and lack of motivation. “I am confident that I am able to complete 1 module of the online training per week even if I do not see positive effects immediately,” was one of the items used in this study. Cronbach alpha was acceptable (alpha=.67).

Recovery self-efficacy was measured with 3 items. The term is used to describe participants’ belief that they can deal with failure and continue to work on the training modules after being nonadherent (ie, after postponing concrete plans and not using the intervention for more than a week). One of the items included here was “I am confident that I can continue working on the training modules even if I postpone my detailed plans several times.” Cronbach alpha was found to be excellent (alpha=.91) in this study.

Risk perception was not included in the model because this factor is likely to be of minor importance in this study sample. Individuals participating in this prevention intervention are assumed to already perceive a high risk for developing a depression which led to help seeking in an Internet-based intervention for depression prevention.

#### Adherence Measure

The number of completed treatment modules in the Internet-based intervention for depression prevention, which ranged from 0 to 7 including the 6 core modules and the booster session 4 weeks after treatment completion, was the primary outcome measure in this study and was tracked automatically by the training platform. To complete a module, participants had to respond to all writing tasks and submit the modules to the system. A module completion score of 0 meant that the participant either did not start the intervention or did not finish the first module.

### Data Analysis

Reliability and descriptive analyses were performed with SPSS 23 (IBM Corp). Structural equation modeling was applied to assess the HAPA model fit and test the hypothesized associations between the model constructs using the Lavaan package in R (The R Foundation). Maximum likelihood parameter estimation was used with robust (Huber-White) standard errors and a scaled test statistic that is (asymptotically) equal to the Yuan-Bentler test statistic [53]. The structural equation model included the latent exogenous variables outcome expectancies, motivational self-efficacy, maintenance self-efficacy, and recovery self-efficacy; the mediating latent variable planning; and the manifest endogenous variables intention and treatment adherence.

The model fit was assessed with the goodness-of-fit indices chi-square (χ^2^), the χ^2^ value relative to its degrees of freedom (χ^2^/df), the root mean square error approximation (RMSEA), the standardized root mean square residual (SRMR), the comparative fit index (CFI), and the Tucker-Lewis Index (TLI). Adequate model fit was indicated by a nonsignificant χ^2^ value, a χ^2^/df ratio between 0 and 2, CFI and TLI values greater than .95, RMSEA value below .06, and SRMR values below .08 [54]. Four planning items were combined into 2 parcels and used as indicators for the variable planning [55]. For the variable maintenance self-efficacy, a single higher order factor was specified.

## Results

### Descriptive Statistics

Participant characteristics at baseline are shown in Table 1; 80.2% (81/101) of the participants were women. Participants had a mean age of 45 years (SD 11.68 ranging from 23 to 75 years) and an above-average level of education (general qualification for university entrance or higher; 83/101, 82.2%). Many participants had prior experience with psychotherapy (43/101, 42.6%), but only a few had taken advantage of health-related trainings (23/101, 22.8%). As indicated by their responses to the CES-D, the participants showed clinically relevant depressive symptoms (mean 26.61, SD 6.51). The results of the clinical interview at baseline showed that participants were, on average, mildly depressed (mean_HRSD_ 13.72, SD_HRSD_ 6.24; mean_QIDS_ 8.18, SD_QIDS_ 3.63).

As shown in Table 2, participants were, on average, characterized by very high motivational self-efficacy (mean 3.64, SD 0.46) and intention (mean 3.51, SD 0.84) to complete the treatment modules. Participants’ expectations concerning the outcome were high (mean 3.21, SD 0.51), and so were the results concerning maintenance self-efficacy (mean 3.36, SD 0.47) and recovery self-efficacy (mean 3.41, SD 0.57). Participants indicated, however, that they had, on average, not made any specific plans when and how they would complete the training (mean 2.44, SD 0.92).

### Adherence Rates

**Figure 2:**
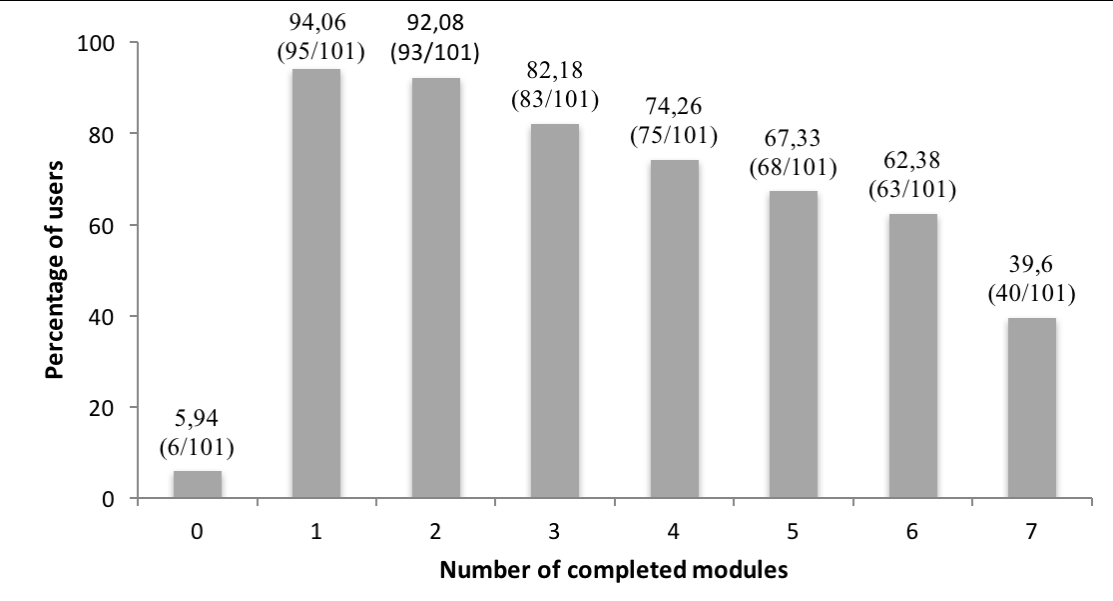
Treatment adherence rates per module.

Figure 2 depicts the number of completed modules. In total, 5.9% (6/101) of the participants did not start the intervention, whereas 62.4% (63/101) completed all 6 core modules, and 39.6% (40/101) completed all 7 modules including the booster session. On average, participants completed 5.12 modules (SD 2.22, range 0-7).

**Table 1:** Baseline characteristics of the study population (N=101).

Characteristic			Intervention group (N=101)
Age, years, mean (SD^a^)			44.57 (11.68)	
**Gender, n (%)**			
	Female		81 (80.2)
	Male		20 (19.8)
**Ethnicity, n (%)**			
	White		78 (77.2)
	Not reported		23 (22.8)
**Relationship, n (%)**			
	Single		26 (25.7)
	Married or cohabited		65 (64.4)
	Divorced or separated		9 (8.9)
	Widowed		1 (1.0)
**Level of education, n (%)**			
	Low^b^		2 (2.0)
	Middle^c^		16 (15.8)
	High^d^		83 (82.2)
**Employment status, n (%)**			
	Employed		89 (88.1)
	Unemployed or seeking work		2 (2.0)
	On sick leave		0
	Not employed		10 (9.9)
**Gross annual income (Euro), n (%)**			
	Low (<10,000)		9 (8.9)
	Middle (10,000-60,000)		69 (68.3)
	High (>60,000)		14 (13.9)
	Not reported		9 (8.9)
**Experience with health-related trainings, n (%)**			
	Yes		23 (22.8)
	No		78 (77.2)
**Experience with face-to-face psychotherapy, n (%)**			
	Yes		43 (42.6)
	No		58 (57.4)
Use of antidepressants, n (%)			7 (6.9)	
CES-D^e^ sum score, mean (SD)			26.61 (6.51)	
HRSD^f^ sum score, mean (SD)			13.72 (6.24)	
QIDS^g^ sum score, mean (SD)			8.18 (3.63)	

^a^SD: standard deviation.

^b^bQualifications below a degree from a German secondary school (Realschule).

^c^Degree by a German secondary school or higher; apprenticeship.

^d^General qualification for university entrance or higher.

^e^CES-D: Center for Epidemiologic Studies Depression Scale.

^f^HRSD: Hamilton Rating Scale for Depression.

^g^QIDS: Quick Inventory of Depressive Symptomatology–Clinician Rating.

**Table 2:** Means and standard deviations for the health action process approach variables at baseline.

HAPA^a^ variables	Mean (SD^b^)	Range
Motivational self-efficacy (2 items)	3.64 (0.46)	3-4
Outcome expectancies (2 items)	3.21 (0.51)	1-4
Intention (1 item)	3.51 (0.84)	1-4
Planning (4 items)	2.44 (0.92)	1-4
Maintenance self-efficacy (4 items)	3.36 (0.47)	2-4
Recovery self-efficacy (3 items)	3.41 (0.57)	1-4

^a^HAPA: health action process approach.

^b^SD: standard deviation.

### Health Action Process Approach Model Fit

**Figure 3:**
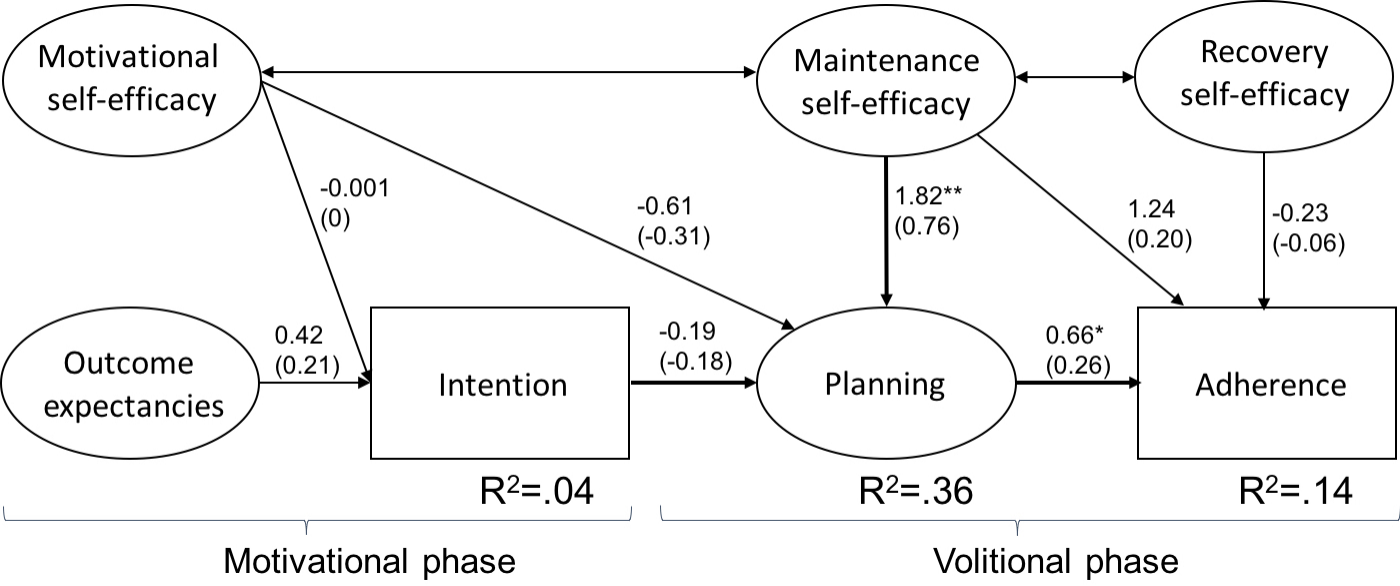
Prediction of treatment adherence with the health action process approach. Note: upper coefficients are unstandardized parameter estimates; lower coefficients (in parentheses) are standardized parameter estimates. *P<.05, **P<.001.

According to the criteria proposed by Schermelleh-Engel et al [54], the HAPA model for treatment adherence yielded a good fit, χ^2^_82_=106.163, *P*=.038, χ^2^/df=1.29, CFI=.96, TLI=.95, RMSEA=.05 (90% CI 0.01-0.08), SRMR=.07, despite the significant results of the chi-square test [54]. Although the chi-square test is often used to evaluate models, this statistic is known to be sensitive to sample size [56]. Figure 3 shows the unstandardized parameter estimates and the standardized parameter estimates (in parentheses). As hypothesized with regard to the volitional phase, higher levels of maintenance self-efficacy predicted more planning (*ß*=0.76, *P*<.001). Planning, in turn, was a significant predictor of treatment adherence at T2 (*ß*=.26, *P*=.04). The other direct associations predicted by HAPA were not significant. Maintenance self-efficacy significantly affected treatment adherence through planning (*b*=1.20, Monte Carlo 95% CI 0.06-2.71). This corresponds to a medium effect of .19 (index of mediation) [57]. The model accounted for 4% of the variance in intention and 36% of the variance in planning. Altogether, 14% of the variance in treatment adherence could be explained.

## Discussion

### Principal Findings

Treatment adherence is challenging for many participants using Internet-based interventions: they intend to participate in an intervention, but they either do not start or do not complete it. This study explored this intention-behavior gap by focusing on motivational and volitional factors as explained by HAPA, adapted with regard to treatment adherence.

As hypothesized, volitional processes clearly affected treatment adherence. More specifically, planning emerged as a significant predictor of treatment adherence. This result indicates that planning might point to an underlying mechanism in adherence in Internet-based interventions. This finding is in line with studies showing that interventions that require participants to engage repeatedly in strategic planning may work because mentally linking intentions to specific contexts increases the likelihood of translating intentions into actions [37,58-61]. As also suggested by the results, it might be easier for individuals with high levels of maintenance self-efficacy to engage in planning than for those with low levels, as the former are confident that they can overcome adherence barriers such as technical problems and the absence of immediate feedback [62,63]. Maintenance self-efficacy, in turn, led to higher treatment adherence through planning. It is, however, also possible that, contrary to the relationship suggested by HAPA, planning might affect maintenance self-efficacy. In this study, we evaluated the proposed relationships between the HAPA variables as suggested by the model. To assess the causality and direction of the effect, a longitudinal intervention study controlling for other influential variables or an experimental design would be necessary.

The motivational profile of individuals who decided to use an Internet-based intervention assumed to be intenders was confirmed by the results showing high levels of motivational self-efficacy, outcome expectancies, and intention of participants in this study. They were, however, not predictive of participants’ treatment adherence. Thus, in line with HAPA, motivational variables seem to no longer exert influence once participants have developed the motivation and intention to participate in an intervention [64]. In contrast, other studies have found the intrinsic motivation of individuals, their belief in their own ability to complete the intervention, and their expectancies regarding treatment outcome to be associated with adherence in Internet-based interventions [7,14,65-68]. Participants of those studies might have been situated in a preintentional stage due to a less elaborated study inclusion process and less detailed information about the expected commitment prior to intervention start, which might have led to the greater influence of intrinsic motivation and expectations on adherence. This assumption is supported by another study which had an elaborated screening process that also found intentions to use an Internet-based intervention not to be related to actual adherence [7].

This study showed that 14% of the variance in treatment adherence could be explained with motivational and volitional processes. In comparison to other studies evaluating predictors of adherence, the amount of explained variance in this analysis is relevant given the small number of variables significantly influencing treatment adherence in this sample and given the restricted variance in variables due to the homogenous study population [12,69]. In a study evaluating the influence of different guidance formats, gender, age, education, symptom-related factors, and hope for improvement, 9.4% of the variance in treatment nonadherence could be explained [12].

While volitional processes seem to be important mechanisms of treatment adherence to Internet-based interventions, future research needs to investigate to which degree other psychological or social variables additionally influence or moderate treatment adherence. Future studies could consider additional HAPA variables such as action control as well as barriers or facilitators that are closely related to the HAPA constructs (eg, perceived social support, perceived support by an eCoach guiding individuals using an Internet-based intervention, or intervention characteristics such as usability). In general, variables may need to be adjusted to the specific context (ie, adherence to and engagement with Internet-based interventions). Moreover, it would be necessary to test whether HAPA variables have an incremental influence beyond sociodemographic, disease-related, and intervention variables.

### Limitations

This study has several limitations. First, treatment adherence was operationalized as the number of completed treatment modules. Completing a module requires working through different writing tasks, but it is difficult to discern whether participants truly engaged with the content of the intervention or applied what they had learned by completing exercises in their day-to-day lives. Second, there might be a subgroup of participants who discontinued using the intervention because they have already attained their personal treatment goal before the end of the training and did not need to complete the entire intervention [70,71]. Thus, higher treatment adherence might not always be related to better treatment outcome. Third, the variance in treatment adherence was restricted, and the sample was very homogeneous. This may have been due to the elaborate screening process, which required prospective participants to be highly motivated to be considered for this study. Thus, these results may underestimate the effects of the HAPA variables on treatment adherence compared to other studies designed to analyze adherence predictors. It is important to note here that the sample consisted of people with depressive symptoms, who show larger volitional deficits than people with other disorders or healthy ones. The results of this study might also in this respect underestimate the effects of HAPA variables on adherence [62,71]. Fourth, participants also received adherence reminders and feedback on demand; both of these elements have been associated with higher adherence rates [12]. Guidance as a potential influence of adherence was, however, a constant factor among all participants in this study because only a few requested feedback. Therefore, guidance is likely to have affected the level of adherence but not to have led to interindividual differences. Fifth, only one adherence measure was included in the analysis. In future studies, different adherence measures need to be used to collect more data on the quality of engagement with an intervention (eg, number of online training diary entries). Moreover, only self-report measures were used, and the reliability was restricted for some of the constructs. The outcome measure (ie, treatment adherence) was assessed objectively. Sixth, due to feasibility limitations, the HAPA variables were only assessed at baseline and therefore cannot account for individual changes in motivational and volitional attitudes at different stages during the treatment process, although these changes might be relevant for treatment adherence. Future studies should therefore include additional measurement points over the course of the intervention to assess motivational and volitional variables concerning individual sessions. Furthermore, negative outcome expectancies and action control were not included in the HAPA model assessed in this study and should be analyzed with regard to treatment adherence in a next step.

### Conclusion and Recommendations for Future Research

As shown in this study, planning was the strongest predictor of treatment adherence and, therefore, should be a key dimension of future Internet-based interventions. Maintenance self-efficacy seems to be a crucial prerequisite in this respect, especially because it allows individuals to overcome potential barriers in the course of treatment. Individuals who had already decided to use the intervention did not seem to need further motivation or positive outcome expectancies at the beginning of this study. Instead, one explanation might be that they might need further support when it comes to detailed planning on how to complete modules on a regular basis to maintain or increase their adherence motivation while using the intervention. Individuals low in planning competences may therefore benefit from identifying possible obstacles and barriers with regard to module completion and develop coping strategies early in the intervention to keep up with module completion. To foster the implementation of these action and coping plans, participants should have the option to formulate if-then plans (eg, “If I do not feel like logging in and completing a module, then I review my treatment goals”). For maintenance self-efficacy purpose, evaluating treatment barriers and developing coping strategies should be repeated throughout the intervention. At the end of each module, participants might also profit from scheduling their next log-in to the intervention for the upcoming week. When individuals do not achieve their personal adherence goals, additional support should be provided to motivate them to retry, choose different coping strategies, or adapt their goals. The value of such tailored strategies in Internet-based preventive interventions to foster volitional competencies regarding treatment adherence in individuals should be evaluated systematically in future research. In this respect, it is also important to identify what works best for whom because different features may have different effects on individuals depending on the motivational state they are in.

Two major strengths of this study were its longitudinal design and objective adherence measure. Due to the limitations outlined above, the main findings of this study will, however, have to be confirmed by future research, which will also have to consider other psychological disorders. Using theoretical frameworks such as HAPA when designing interventions and conducting research is important because it allows researchers to test a given theory’s proposed relationships, and, if these can be confirmed, this approach could provide a blueprint for effective future interventions.

## Appendix

Multimedia Appendix 1 Correlation matrix and health action process approach item scores.

## Abbreviations

AES: Advanced Encryption Standard
CES-D: Center for Epidemiological Studies Depression Scale
CFI: comparative fit index
eCoach: electronic coach
HAPA: health action process approach
HRSD24: Hamilton Rating Scale for Depression
QIDS-CR16: Quick Inventory of Depressive Symptomatology–Clinician Rating
RCT: randomized controlled trial
RMSEA: root mean square error approximation
SRMR: standardized root mean square residual
TLI: Tucker-Lewis Index
