# Supplementary material for: Turning Good Intentions Into Actions by Using the Health Action Process Approach to Predict Adherence to Internet-Based Depression Prevention: Secondary Analysis of a Randomized Controlled Trial
Source: J Med Internet Res. 2018 Jan 11;20(1):e9. doi: 10.2196/jmir.8814 (PMC5785685; doi:10.2196/jmir.8814)
Supplement: Multimedia Appendix 1 [file jmir_v20i1e9_app1.pdf]

Appendix Tables

Table 3. Means and standard deviations for the HAPA variables per item at baseline.

**HAPA variables per item**	Mean	SD[a]	Range
**Motivational self-efficacy**			
I am confident that I am able to ...			
complete one module of the online training per week even if there might be problems.	3.66	0.48	3-4
complete all six modules of the online training and the booster session four weeks after completion of the training even if there might be problems.	3.62	0.49	3-4
**Outcome expectancies**			
If I complete one module of the online training per week …			
I will learn to cope with stress.	3.29	0.57	1-4
I will become more resilient in my everyday life.	3.14	0.60	1-4
**Intention**			
I intend to complete …			
all six modules of the online training and the booster session four weeks after completing the training.	3.50	0.84	1-4
**Planning**			
I have already made detailed plans …			
when to complete the modules.	2.25	1.07	1-4
how often I will work on the modules during the week.	2.30	1.04	1-4
how to complete the modules despite my other obligations and interests.	2.62	0.98	1-4
how to continue working on the modules even if something comes up.	2.60	1.00	1-4
**Maintenance self-efficacy**			
I am confident that I am able to complete one module of the online training per week …			
even if I do not see positive effects immediately.	3.51	0.54	2-4
even if I would like to do something else.	3.39	0.63	1-4
even if I experience technical problems with the computer or with the internet.	3.37	0.64	2-4
even if it is hard for me to do so.	3.19	0.82	1-4
**Recovery self-efficacy**			
I am confident that I can continue working on the training modules ...			
even if I postpone my detailed plans several times.	3.42	0.62	1-4
even if I am not able to pull myself together sometimes.	3.46	0.61	1-4

even if I have not worked on a module for several days.	3.37	0.64	1-4

Note. [a]SD = standard deviation

Table 4. Correlation matrix of the HAPA variables with treatment adherence.

	**Adh**	**Mot SE**	**Out Expt**	**Intent**	**Plan**	**Maint SE**	**Rec SE**
**Adh**	1	.22*	.11	-.09	.31**	.17	.15
**Mot SE**	.22*	1	.16	.04	.18	.45**	.39**
**Out Expt**	.11	.16	1	.14	.35**	.24*	.11
**Intent**	-.09	.04	.14	1	-.06	.14	.14
**Plan**	.31**	.18	.35**	-.06	1	.39**	.21**
**Maint SE**	.18	.45**	.24*	.14	.39**	1	.59**
**Rec SE**	.15	.39**	.11	.14	.21*	.59**	1

Note. *P< .05, **P < .01. Adh = Adherence, Mot SE = Motivational self-efficacy, Out Expt = Outcome expectancies, Intent = Intention, Plan = Planning, Maint SE = Maintenance self-efficacy, Rec SE = Recovery self-efficacy
